# Sperm hyperactivation in the uterus and oviduct: a double-edged sword for sperm and maternal innate immunity toward fertility

**DOI:** 10.1590/1984-3143-AR2024-0043

**Published:** 2024-08-16

**Authors:** Ihshan Akthar, Mohamed Samy Yousef, Alireza Mansouri, Masayuki Shimada, Akio Miyamoto

**Affiliations:** 1 Global Agromedicine Research Center (GAMRC), Obihiro University of Agriculture and Veterinary Medicine, Obihiro, Japan; 2 Department of Theriogenology, Faculty of Veterinary Medicine, Assiut University, Assiut, Egypt; 3 Graduate School of Integrated Sciences for Life, Hiroshima University, Higashi-Hiroshima, Japan

**Keywords:** sperm, hyperactivation, uterus, oviduct, Toll-Like Receptor 2

## Abstract

In cattle, artificial insemination (AI) is a technique that allows breeding by depositing frozen-thawed and extended semen into the female reproductive tract. The semen contains sperm with various motility patterns including dead, progressive and hyperactivated. Sperm hyperactivation is high amplitude, asymmetrical beating of sperm tail which usually occurs in the oviduct as part of the capacitation process, but it can also be induced by cryopreservation. After insemination, sperm enter the uterine glands and trigger a pro-inflammatory response in the uterus. Hyperactivated sperm, stimulated by sperm-Toll-like receptor 2 (TLR2), penetrates the mucus and uterine glands more efficiently and enhances the immune response. This facilitates the clearance of excess and dead sperm from the uterus. Some sperm escape the immune response and reach the oviduct either before or after the immune response is initiated. In the oviduct, sperm bind to the epithelium and form a reservoir. This triggers an anti-inflammatory response and preserves the fertilization potential of sperm. Hyperactivation facilitates sperm detaching from the epithelium, swimming through the viscous mucus and cumulus cells, and penetrating the egg's zona pellucida. Sperm-TLR2 activation enhances Ca^2+^-influx and acrosome reaction, which enables sperm to penetrate and fertilize oocytes during in vitro fertilization. Altogether, post-AI in cattle, sperm and maternal immunity interact differentially depending upon the site of sperm hyperactivation – whether it occurs within the uterus or oviduct. Specifically, hyperactivated sperm that enter the uterus after AI or are triggered via sperm-TLR2 activation or other stimuli contribute to sperm-induced uterine inflammation. Such hyperactivated sperm may impede their capacity to ascend to the oviduct. Conversely, sperm that become hyperactivated within the oviduct modulate their interactions with the oviduct and oocytes, which is pivotal during fertilization process. Indeed, the location and timing of sperm hyperactivation partially via TLR2 activation are critical determinants of their different influence on fertility.

## Introduction 

In mammals, reproduction is a vital process to produce offspring, and it is usually achieved by the fusion of a single sperm with an oocyte. However, during natural mating or artificial insemination (AI), a massive number of sperm are introduced into the female reproductive tract (FRT) to increase the probability of fertilization.

Natural mating involves the introduction of semen, containing a large number of sperm and seminal plasma (SP) components into the FRT. In cattle and humans, semen is deposited in the vagina and from there, sperm navigate to the uterine lumen (Sobrero and Macleod., 1962; López-Gatius, 2000). In rodent species semen is deposited in the vagina and most of the sperm pass rapidly into the uterine cavity along with the SP (Carballada and Esponda, 1997). In species such as pigs, semen is directly deposited into the uterine cavity (Hunter, 1981). Notably, the deposition site of semen during natural mating varies across species, influencing the subsequent migration of sperm within the FRT.

AI is a method of breeding animals, especially cattle, by placing semen, diluted with an extender that reduces the amount of SP, directly into the uterus (López-Gatius, 2000; Vishwanath, 2003). After AI, the sperm cells have to travel through the FRT and encounter various anatomical structures and physiological factors, such as the uterus, utero-tubal junction, the oviduct and their immune system. These immune interactions influence sperm movement and survival and ultimately determine the success of fertilization.

In this review, we discuss the regulatory mechanisms by which sperm modulate inflammatory responses within the bovine reproductive tract, especially the uterus and oviduct, and the subsequent impact on reproductive efficiency, with a particular focus on AI. We emphasize the role of sperm hyperactivation, a phenomenon associated with AI in cattle, in modulating the above inflammatory signals. We employed a range of approaches, in vivo, ex vivo, in vitro and computational models, to clarify the physiological and molecular mechanisms involved in these processes.

## Sperm dynamics in the bovine FRT after AI

AI is the most established and prevalent assisted reproductive technology in cattle. It is a technique that involves diluting the ejaculated semen with an extender to increase the number of doses that can be obtained from a single ejaculation (Vishwanath, 2003). The diluted semen is filled into straws that are then frozen and stored in liquid nitrogen until needed. At the time of insemination, the straws are thawed and the content, with the extender, a massive number of live and dead sperm along with a negligible amount of SP is directly deposited into the uterine body of the cow (López-Gatius, 2000; Vishwanath, 2003). One should note that the dilution process reduces the amount of SP that enters the uterus along with the sperm. However, SP also forms a coating around the sperm surface, which may influence sperm function in the FRT (Suarez and Pacey, 2006; Suarez, 2016).

After AI or mating, a substantial portion of sperm is lost from the vagina through fluid backflow (Dobrowolski and Hafez, 1970; Hawk, 1987; Marey et al., 2023). The remaining sperm in the uterus migrate to the oviduct, influenced by the uterine environment. We recently showed that sperm are rapidly moved from the uterine body to either the uterine horn or the vagina within 1h after AI. Only a few sperm are found in the uterine horn or vagina 6h after AI, and none are detected 10h after AI (Marey et al., 2023). This suggests that sperm are rapidly transported in both directions from the site of semen deposition after AI. In cattle, the rate of sperm passage through the uterus is poorly understood. After natural mating, sperm take about 6-8h to reach the oviduct and fertilize the ovum (Wilmut and Hunter, 1984). Sperm are also detected in the oviducts within 1h after natural mating or AI (Hawk, 1987), but their role in fertilization is unclear.

As sperm moves through the uterus towards the oviduct, they engage in complex interactions with the maternal environment and the immune system. This journey is also essential for ensuring sperm capacitation, a prerequisite for successful fertilization. Sperm capacitation involves a series of biochemical and biophysical changes in the sperm membrane. These changes are crucial as they prepare the sperm for hyperactivation, the acrosome reaction (AR) and ultimately, penetration of the oocyte (Suarez, 2008). Capacitation is modulated by various factors, including the FRT’s secretions and the methodologies employed for sperm preservation (Gillan et al., 1997; Bailey et al., 2000; Cormier and Bailey, 2003).

Hyperactivation, a key step in the capacitation process, helps sperm to overcome the barriers of the female tract and reach the oocyte. There is evidence that hyperactivated sperm cannot traverse the uterotubal junction (Gaddum-Rosse, 1981). This suggests that the oviduct is a suitable site for hyperactivation to ensure successful fertilization. However, freezing and thawing sperm (i.e., cryopreservation) can also trigger hyperactivation prematurely (Gillan et al., 1997; Bailey et al., 2000; Cormier and Bailey, 2003; Akthar et al., 2023). Thus, after AI, the hyperactivated portion of sperm that is exposed to the uterus could affect sperm-uterus communication. Moreover, these hyperactivated sperm may lose their ability to reach and interact with the oviductal environment. On the other hand, the sperm that hyperactivates later in the oviduct could regulate the interaction with the oviduct and oocytes during fertilization. Therefore, understanding the role of hyperactivation in the female tract is crucial for improving the success rate of AI in cattle.

## Sperm hyperactivation

Sperm hyperactivation is a type of sperm motility characterized by high amplitude, asymmetrical flagellar bending (Yanagimachi, 1970). Both hyperactivation and AR are required for fertilization. For detailed reviewed information on sperm hyperactivation, please refer to Suarez (2008). Hyperactivated sperm can be detected by computer-assisted sperm analysis (CASA) systems, which use criteria such as curvilinear velocity (VCL), amplitude of lateral head displacement (ALH) and path linearity (LIN) to measure sperm movement (Mortimer et al., 1998; Marquez and Suarez, 2007).

The initiation and maintenance of sperm hyperactivation depends on Ca^2+^ influx primarily through CatSper channels and possibly Ca^2+^ release from the nuclear envelope (Ho and Suarez, 2001; Ho and Suarez, 2003; Marquez and Suarez, 2007). It also requires high pH and ATP levels (Ho et al., 2002; Marquez and Suarez, 2007). In addition, Toll-like receptor 2 (TLR2) has been shown to mediate both hyperactivation and AR in bovine sperm (Ma et al., 2022; Akthar et al., 2023). The exact physiological signals that initiate hyperactivation in vivo are still unclear.

Sperm encounter viscoelastic fluids in different compartments of the FRT during their passage. These fluids include estrous-cervical-mucus (Tung et al., 2015), endometrial mucus (Akthar et al., 2020), oviduct fluid (Suarez et al., 1997), the matrix of the cumulus oophorus and zona pellucida (ZP) (Dunn and Picologlou, 1976; Kim and Kim, 2013). Hyperactivation enhances the ability of sperm to penetrate highly viscoelastic media, as shown by previous studies (Suarez et al., 1991; Suarez and Dai, 1992). In our recent work, we demonstrated that hyperactivated bull sperm can effectively penetrate estrous-uterine-mucus (Akthar et al., 2023). These suggest that hyperactivation may modulate the interaction and responses between sperm and FRT fluids, which could have implications for fertilization success.

The following sections will explore how sperm, especially those with high motility, affect the interactions with the uterus and oviduct, focusing on our latest findings in bovine species.

## Sperm-uterine interactions trigger a pro-inflammatory immune response

The uterus performs several vital functions for reproduction. The endometrium, nourishes and houses a fertilized egg until the fetus is ready for parturition (Spencer et al., 2005). The uterus also facilitates the movement of sperm to the oviduct. Additionally, the sperm-triggered uterine immune response helps remove excess and dead sperm, and other foreign substances from the FRT to support embryo implantation, and also prevent possible infections derived from AI or semen (Akthar et al., 2021).

### Sperm interaction with the uterine epithelium

After entering the uterus, sperm encounter the uterine microenvironment, which consists of a thick, clear, viscoelastic mucus layer that covers the endometrial epithelium. Sperm also interact with the uterine microarchitecture and the uterine innate immune system, which influences their survival and fertilization potential.

We developed an ex vivo model to investigate the real-time interactions of sperm with the uterus in cattle (Akthar et al., 2020). The endometrial tissue disks which contained surface and glandular epithelium, underlying stroma and a mucus coating were used in this model. Sperm were seen glided over the mucus layer and entered the uterine glands, without attaching to the surface epithelium. This suggested that the uterine gland is a microenvironment where sperm reside and interact with the endometrial cells. Similar sperm-uterine interactions were reported in several other mammalian species, such as dogs, rabbits and swine (Doak et al., 1967; Koyama et al., 1986; Rijsselaere et al., 2004). These support the notion that the uterine gland is a niche where sperm are retained in the uterus across different species.

### Sperm trigger a pro-inflammatory immune response in the uterus

Our in vitro, ex vivo and in vivo experiments demonstrated that sperm trigger a pro-inflammatory immune response in the bovine uterus. In the in vitro model, sperm elicited an inflammatory response in bovine endometrial epithelial cells (BEECs), as evidenced by increased mRNA expression of pro-inflammatory cytokines such as interleukin 8, tumor necrosis factor-alpha (TNFA), interleukin 1B (IL1B), and nuclear factor-kappa B2 as well as prostaglandin E synthase (PGES). Sperm also downregulated mRNA expression of the anti-inflammatory cytokine, transforming growth factor-beta 1 (TGFB1). Furthermore, medium from sperm-BEECs co-culture enhanced sperm phagocytosis by polymorphonuclear neutrophils (PMNs) (Elweza et al., 2018). These results indicate that live bull sperm trigger an acute uterine inflammatory response in vitro.

In the ex vivo model, we found that active sperm enter the uterine glands and induce an inflammatory response, as evidenced by increased mRNA expression of pro-inflammatory cytokines (Akthar et al., 2020). In contrast, heat-inactivated (immotile) sperm did not enter the uterine glands or trigger the inflammatory response. Sperm also increased TNFA protein expression in the glandular epithelium. In addition, PMNs were present in the glands among the clusters of sperm and some sperm appeared to bind to the PMNs. However, PMNs were not detected in the uterine glands in the absence of sperm. These results indicate that the initial route of PMNs into the uterine cavity is through the uterine glands. The presence of PMNs along with sperm in uterine glands indicated a possible uterine innate immune activation in response to sperm. These phenomena revealed that the uterine gland acts as a sensor for sperm to trigger the uterine innate immune cascade.

In the in vivo experiments, we investigated the local and systemic maternal immune responses of sperm (Marey et al., 2023). The PMNs infiltrated the uterine body and horn 6h after AI but decreased at 10h and disappeared at 24h. This rapid and transient PMN influx should facilitate the clearance of excess, dead and damaged sperm from the uterus. However, it may also compromise the survival of normal sperm. Therefore, the swift transport of viable sperm to the oviduct before the arrival of PMNs appears to be crucial for successful fertilization. Sperm also stimulated the pro-inflammatory responses in PMNs and peripheral blood mononuclear cells. These results suggest that sperm elicit a transient pro-inflammatory reaction both locally in the uterus and systemically in the circulatory blood after AI.

These comprehensive experiments demonstrated that sperm stimulate the uterine glands and trigger the transient innate immune cascade in the uterus after AI in cattle.

### Molecular mechanism of sperm-uterine immune interaction

**Figure 1:**
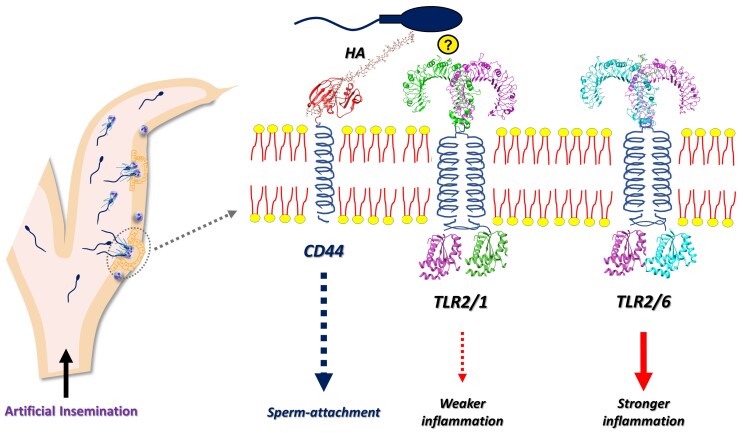
The schematic illustration for the molecular mechanism of sperm-uterine immune interactions in cattle. Hyaluronan (HA), an abundant endogenous molecule in the bovine endometrium, enhance sperm attachment, mainly through its main receptor CD44, in the endometrial epithelium. In the bovine endometrium, activation of the TLR2/1 pathway induces a weaker inflammatory response while the TLR2/6 pathway induces a stronger inflammation. Notably, sperm trigger a weak inflammatory response in the bovine endometrium by activating the TLR2/1 signaling pathway. This weak inflammation enables uterine clearance without causing tissue damage. The specific molecules that make a bridge between TLR1 and TLR2 are unknown. Altogether, HA-CD44 interaction facilitates sperm-endometrium adhesion, while other unknown molecules likely trigger TLR2/1 heterodimerization to induce weak uterine inflammation in cattle.

The molecular mechanism of sperm-uterine immune interaction is summarized in Figure 1. We demonstrated that TLR2, a type of TLR, is associated with sperm-uterine interactions in cattle. TLRs are membrane proteins that sense pathogen-associated molecular patterns and activate innate immune responses (Mogensen, 2009). The sperm induces TLR2 expression in the uterine epithelium, especially in the glandular cells, suggesting that sperm communicates with the uterus via TLR2 (Akthar et al., 2020; Elesh et al., 2021; Mansouri et al., 2023). The blocking of TLR2 in BEECs prevented the sperm-induced activation of pro-inflammatory genes (Ezz et al., 2019). In the ex vivo model, the blocking of endometrial TLR2 reduced the sperm numbers in the uterine glands and inhibited the increase of TNFA expression (Akthar et al., 2020). These findings revealed that sperm utilizes the endometrial TLR2 to regulate the uterine immune cascade in cattle.

However, activation of the TLR2 system depends on the formation of heterodimers with other TLRs (TLR1 and TLR6). These heterodimers can trigger distinct inflammatory responses in the bovine endometrium. To elucidate the TLR2 pathway activated by sperm in the bovine endometrium, we utilized two synthetic lipopeptides: Pam3CSK4 (PAM3; TLR2/1 agonist) and Pam2CSK4 (PAM2; TLR2/6 agonist) (Mansouri et al., 2023). The sperm induced the expression of the TLR2 gene and protein, along with TLR1, but not TLR6 especially in the surface and glandular epithelium. Furthermore, the sperm and PAM3 induced comparable and lower pro-inflammatory responses in the bovine endometrium than PAM2. This implies that sperm trigger a weak inflammatory response in the bovine endometrium by activating the TLR2/1 signaling pathway. In contrast, activating TLR2/6 signaling in the endometrium could induce a stronger and longer inflammation, possibly leading to tissue damage (Monlish et al., 2020; Mansouri et al., 2023). Thus, it seems that sperm exploit TLR2/1 heterodimerization in the bovine endometrium to induce weak and short-term inflammation as a defensive strategy.

Sperm upregulates the cluster of differentiation (CD44) expression in the endometrium, indicating its importance for sperm-endometrium interaction (Elesh et al., 2021). We examined the role of hyaluronan (HA), a potential ligand for CD44 (Vuorio et al., 2017) and a regulator of TLR2 (Erridge, 2010). The in-silico analysis showed that HA binds to CD44 with higher affinity, but not to TLR2, suggesting that HA does not affect TLR2/1 heterodimerization (Ezz et al., 2023). Further, HA enhances sperm attachment to the BEECs and upregulates the expression of pro-inflammatory genes. However, HA alone does not affect BEEC inflammation (Ezz et al., 2023). Thus, it is likely that HA-CD44 interaction facilitates sperm-endometrium adhesion, while other unidentified molecules trigger TLR2/1 heterodimerization to induce weak uterine inflammation in cattle.

## Hyperactivation facilitates sperm-triggered uterine inflammation: sperm-TLR2 as a regulator

**Figure 2:**
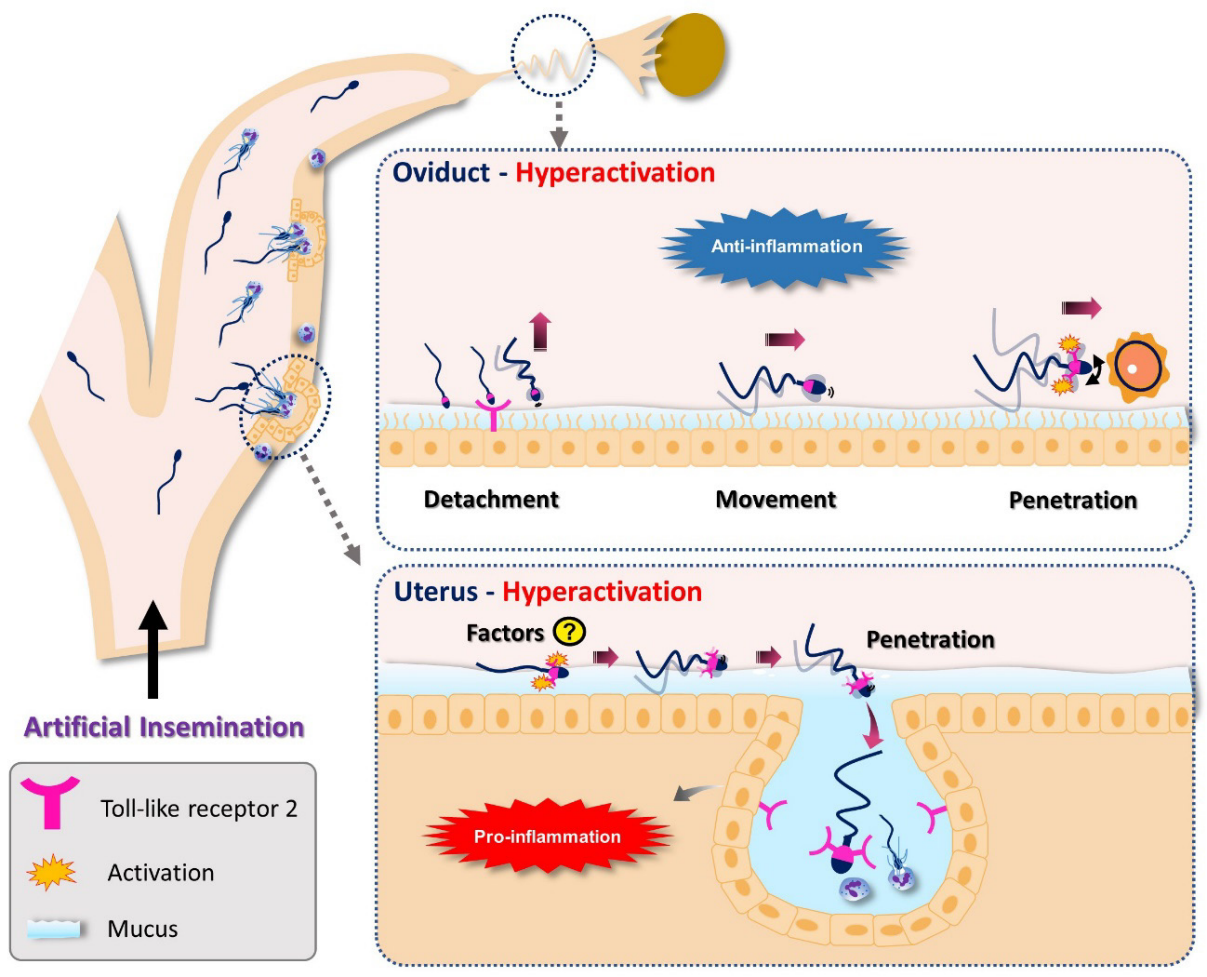
The schematic illustration of the role of hyperactivated sperm in the uterus and oviduct after artificial insemination (AI) in cattle. Hyperactivation usually occurs in the oviduct as part of the capacitation process, but it can also be induced by cryopreservation. In the uterus, hyperactivated sperm, stimulated by sperm-TLR2, penetrates the mucus and uterine glands more efficiently and enhances the pro-inflammatory immune response. The endogenous ligands/triggers (factors) that activate sperm-TLR2 and hyperactivation in the uterus remain unidentified. The endometrial TLR2 is involved in the innate immune response by the uterus to sperm. Altogether, hyperactivated sperm that enter the uterus after AI or are triggered via sperm-TLR2 activation or other stimuli contribute to sperm-induced uterine inflammation. In the oviduct, sperm bind to the epithelium of the isthmus and ampulla. Sperm binding to the ampullary epithelium is partly regulated by ampullary-TLR2. Sperm binding to the oviductal epithelium triggers an anti-inflammatory immune response and preserves the fertilization potential of the sperm. Hyperactivation facilitates sperm detaching from the epithelium, swimming through the viscous mucus and cumulus cells, and penetrating the zona pellucida of the egg. Sperm-TLR2 activation also enhances calcium-influx, hyperactivation and acrosome reaction, which enables sperm to penetrate and fertilize oocytes. In a nutshell, after AI in cattle, sperm and maternal innate immunity interact differentially depending on whether hyperactivated sperm occurs in the uterus or the oviduct.

The possible role of hyperactivation in the uterus is explored below (Figure 2). We recently investigated how hyperactivated sperm, particularly induced by sperm-TLR2 activation affects the interaction of sperm with bovine endometrium, focusing on their penetration of the endometrial mucus and uterine glands (Akthar et al., 2023). We confirmed that bull sperm express TLR2, as reported in the other species (Palladino et al., 2008; Fujita et al., 2011; Hu et al., 2016; Zhu et al., 2016) and showed that it is localized in the posterior segment of the sperm head.

The CASA results revealed that sperm-TLR2 regulates hyperactivation. The hyperactivated sperm in cattle was defined as those with VCL ≥ 200µm/s, ALH ≥ 3µm and LIN ≤ 40% (Akthar et al., 2023). We observed that a portion of sperm was hyperactivated without any stimuli, which could influence their interaction with the endometrium after AI. Activation of sperm-TLR2 by a specific agonist increased the proportion of hyperactivated sperm, while TLR2 blockage by an antagonist reduced it. The exact mechanism of sperm-TLR2 in hyperactivation is unclear. However, it was previously shown that sperm-TLR2 enhances Ca^2+^ influx (Ma et al., 2022). Therefore, sperm-TLR2 may modulate the activity of sperm CatSper channels, which in turn mediate hyperactivation.

We investigated how sperm-TLR2, which regulates hyperactivation, affects sperm penetration of the endometrial mucus layer to reach the uterine glands (Akthar et al., 2023). The percentage of sperm that penetrated the estrous-uterine-mucus was calculated by layering sperm suspensions over them. The motility patterns of sperm that penetrated this mucus were also assessed. Activation of sperm-TLR2 enhanced sperm hyperactivation and penetration of the estrous-uterine-mucus. In contrast, blocking of sperm TLR2 had the opposite effects. Furthermore, the sperm that penetrated the estrous-uterine-mucus after TLR2 activation showed higher motility and hyperactivation. These results suggest that sperm-TLR2 activation enhances sperm hyperactivation and endometrial mucus penetration.

Then the ex vivo model was used to examine the impact of sperm-TLR2 on sperm penetration into the uterine glands. The TLR2 activation enhanced sperm hyperactivation and motility, allowing more sperm to enter the uterine glands and inducing a pro-inflammatory response. In contrast, TLR2 blockage reduced sperm hyperactivation, resulting in fewer sperm entering the uterine glands and eliciting a mild inflammatory response. These results indicated that activation of sperm-TLR2 facilitates sperm entering into the uterine glands to trigger the uterine inflammatory cascade.

Altogether, these results showed that sperm-TLR2 activation leads to sperm hyperactivation, which enables them to penetrate the endometrial mucus and uterine glands, initiating the uterine inflammatory cascade (Akthar et al., 2023). The nature of the endogenous ligands/triggers that activate sperm-TLR2 and hyperactivation in the uterus is unknown, but they could be derived from the uterine microbiota (Appiah et al., 2020; Ballas et al., 2021). The above results suggested that hyperactivated sperm that enter the uterus after AI or hyperactivation in response to other stimuli in the uterus, play a role in triggering the uterine inflammatory cascade. These hyperactivated sperm are unlikely to reach the oviduct and fertilize the oocyte but rather induce an innate immune response in the uterus. On the other hand, linear progressive motile sperm in the uterus, which have a higher fertilizing potential, migrate to the oviduct and participate in fertilization (Shalgi et al., 1992; Ferraz et al., 2014). In a nutshell, sperm hyperactivation by sperm-TLR2 activation or other stimuli such as the freezing-thawing process of sperm used for AI contributes to the induction of the inflammatory cascade in the uterus.

## Sperm-oviduct interactions trigger an anti-inflammatory immune response

The oviduct mainly contains segments such as the isthmus, ampulla, and infundibulum. It performs several functions essential for reproduction, such as aiding the transport of ovum and sperm to the site of fertilization, providing a suitable environment for sperm capacitation and survival, facilitating the fusion of gametes and nourishing and transporting the early embryo to the uterus (Ellington, 1991).

### Sperm interaction with the oviductal epithelium

When the sperm reach the oviduct, many of them bind by their heads to the beating cilia of the isthmus to form a sperm storage reservoir (Suarez et al., 1997; Ardon et al., 2016). In bovine species, this binding is mediated by the bovine seminal plasma proteins called Binder of SPerm (BSP) BSP1, BSP3 and BSP5 that coat the sperm surface (Gwathmey et al., 2003, 2006). Bovine sperm surface proteins bind to annexin proteins A1, A2, A4 and A5 on the oviduct, which may help to retain sperm in the oviductal reservoir (Ignotz et al., 2007). Holding sperm in the storage reservoir helps them to survive, undergo capacitation and exhibit characteristics of hyperactivation (Pollard et al., 1991; Gualtieri and Talevi, 2003).

During the pre-ovulatory period, sperm migrate from the isthmus to the ampulla (Coy et al., 2012). The ampullary lumen has a more complex structure than that of the isthmus in cattle, with primary and secondary mucosal folds that create a large surface area for sperm-epithelium interactions (Suarez et al., 1997; Yániz et al., 2000). These suggest that sperm may benefit from prolonged contact with the ampullary epithelium. We used an explant model to investigate the sperm-ampullary interactions (Morillo et al., 2020). The ampullary primary mucosal folds were incubated with heparin (a known capacitation inducer)-treated or non-treated sperm. Both sperm bound to the ciliated epithelium in similar numbers and aligned themselves with the ciliary beat direction. The receptors on the ampullary surface may modulate sperm-ampullary binding. We examined the involvement of TLR2, in sperm-ampullary interactions (Morillo et al., 2020). The addition of the TLR2 blocker to the heparin-treated or non-treated sperm-explant co-incubations reduced sperm attachment. Further, the addition of the blocker to the co-incubation inhibited TLR2 protein expression. These suggested that the oviductal-TLR2 is at least partly involved in sperm binding to the ampullary epithelium.

### Sperm trigger an anti-inflammatory immune response in the oviduct

In the bovine isthmus and ampulla, the anti-inflammatory cytokines TGFB1 and interleukin 10 are constantly localized throughout the estrous cycle (Yousef et al., 2016). The attachment of sperm to the bovine oviductal epithelial cells (BOECs) further stimulated the mRNA expression of the above anti-inflammatory cytokines. Moreover, sperm trigger PGE2 secretion (Kodithuwakku et al., 2007) which in turn suppresses the pro-inflammatory cytokines (Yousef et al., 2016) in BOECs. Further, the stimulation of PMNs by BOECs-conditioned media suppressed sperm phagocytosis by PMNs and the luteinizing hormone (LH)-stimulated BOECs-conditioned media further suppressed the phagocytosis. Here the LH triggered the BOECs to secrete PGE2, which might be the factor that reduced the phagocytic function (Marey et al., 2013). Moreover, the media conditioned with BOECs decreased the mRNA expression of TNFA and induced the PGES and anti-inflammatory cytokines in PMNs (Yousef et al., 2016). Altogether the findings indicate that in the physiological state, the oviduct is in a stable anti-inflammatory phase and the sperm-oviductal crosstalk further strengthens this state towards a favorable environment for sperm survival at least until fertilization.

## The impact of hyperactivated sperm in the oviduct

Hyperactivation enables sperm to detach from the oviductal epithelium, navigate through the viscous mucus and cumulus matrix, and penetrate the ZP surrounding the egg. The role of hyperactivation in the oviduct is discussed in-depth below (Figure 2).

### Hyperactivation enhances sperm detachment from the oviductal epithelium

Sperm detachment from the oviductal epithelium, where sperm are temporarily stored after mating, is a crucial step for fertilization. This process is facilitated by two changes during capacitation: modification of cell surface proteins and hyperactivation. The former reduces the binding affinity of sperm for oviductal receptors, while the latter enables sperm to overcome the viscous environment of the female tract and pull away from the epithelial surface (Simons et al., 2014). Hyperactivation is essential for sperm detachment, as shown by several studies. In mice, hyperactivation is involved in the release of sperm from the sperm reservoir at the isthmus (DeMott and Suarez, 1992; Chang and Suarez, 2012). Hyperactivated sperm exhibit a rocking motion that enables them to detach from the cilia on the epithelial surface (Chang and Suarez, 2012). Mouse sperm that lack functional CatSper channels, which are required for hyperactivation, fail to detach from the oviduct (Ho et al., 2009). In porcine, hyperactivation is necessary and sufficient to detach sperm from specific glycans on the oviduct epithelium that immobilizes sperm in the isthmus (Sharif et al., 2022).

In cattle, hyperactivation also plays a role in sperm detachment from the oviductal epithelium (Ardon et al., 2016). Heparin, which blocks BSP-epithelium interactions and reduces the binding affinity of sperm for epithelial cells could detach sperm from the isthmus but not the ampulla. However, when hyperactivation was induced along with heparin, sperm could be released from both regions. These suggest that hyperactivation enhances sperm detachment by overcoming the adhesion to the oviductal epithelium. Altogether, these findings indicate that hyperactivation plays a significant role in sperm detachment from the oviductal epithelium, and thus in the success of fertilization.

### Hyperactivation facilitates sperm migration towards oocytes

Hyperactivation is essential for sperm to traverse the oviduct, where they face various physical and chemical obstacles. One of these obstacles is viscoelastic mucus that is secreted by the oviductal epithelium in cattle and other mammals (Jansen, 1980; Suarez et al., 1992; Suarez et al., 1997). Hyperactivation facilitates the sperm’s progress through this mucus in the oviductal lumen (Suarez and Dai, 1992; Suarez et al., 1992). Hyperactivation can also be influenced by chemotactic factors, such as progesterone released by the cumulus cells surrounding the oocyte, which guide sperm to the site of fertilization as demonstrated in some mammalian species (Harper et al., 2004; Guidobaldi et al., 2008). Therefore, hyperactivation is a key mechanism for sperm navigation and movement through the oviduct to the oocytes in mammals.

### Hyperactivation mediates sperm penetration to cumulus-oocyte complexes (COCs)

Sperm need to overcome the highly viscoelastic barrier of the cumulus oophorus, a cluster of cells that surround the oocyte, to reach the ZP and fertilize the egg. The main component of the cumulus matrix is hyaluronan, which confers resistance and elasticity to the cumulus cells. Sperm can partially degrade the hyaluronan network by releasing hyaluronidase from their acrosome, but this is not sufficient for sperm penetration. Sperm also need to exhibit hyperactivated motility that enhances their swimming force and ability to navigate through the cumulus oophorus (Suarez and Dai, 1992).

Hyperactivation is also required to penetrate the thick and protective layer that surrounds the egg, known as ZP. By undergoing hyperactivation, the sperm can increase its motility and generate enough force to break through the ZP and fuse with the egg membrane. Hyperactivated sperm penetrates the ZP more effectively than non-hyperactivated sperm (Katz and Yanagimachi, 1981; Stauss et al., 1995; Quill et al., 2003). The large tail undulations of hyperactivated sperm could provide a thrust against objects, such as the cumulus oophorus and the ZP (Katz et al., 1978) to efficiently penetrate them.

### TLR2 regulates sperm interaction with COCs

TLR2 has been shown to play a significant role in fertilization, especially during in vitro fertilization (IVF). In mice, the sperm-generated hyaluronan fragments act as endogenous ligands for TLR2 and TLR4 on cumulus cells, triggering the production of certain cytokines/chemokines that facilitate sperm capacitation and fertilization. This indicates that the TLR2/4 pathway mediates the communication and coordination between sperm and COCs (Shimada et al., 2008).

Recently, we demonstrated the role of sperm-TLR2 in sperm-oocyte interactions using an IVF model in cattle (Ma et al., 2022). The results showed that activation of sperm-TLR2 enhances the cleavage and blastocyst rates in both COCs and cumulus-free oocytes, but not in ZP-free oocytes. In contrast, TLR2 blockage reduced the cleavage and blastocyst rates in both COCs and cumulus-free oocytes, but not in ZP-free oocytes. Observing fluorescence images of sperm-ZP interactions revealed that TLR2 activation or blockage enhanced and reduced, respectively both the binding and penetration abilities of sperm to ZP. Furthermore, TLR2 regulated the AR in ZP-attached sperm, suggesting that sperm-TLR2 plays a physiological role in the sperm-oocyte crosstalk via regulating ZP-triggered AR. Moreover, we found that sperm-TLR2 activation also induces sperm hyperactivation (Akthar et al., 2023). Hyperactivation is regulated by several factors, one of which is Ca^2+^ as discussed in the previous sections. We observed that TLR2 activation increased the sperm response to Ca^2+^, while TLR2 blockage decreased it. Altogether these findings suggested that sperm-TLR2 is involved in hyperactivation and AR induction, which enables sperm to penetrate and fertilize oocytes during the IVF at least in cattle.

## Final considerations - the timing of hyperactivation decides the fate of sperm in FRT

Sperm interaction with the mucosal epithelium of the bovine uterus and oviduct triggers distinct immune responses that vary by location and timing. In the uterus, sperm elicit a pro-inflammatory response aimed at clearing surplus sperm, whereas in the oviduct, they induce an anti-inflammatory response that promotes fertilization. Notably, after AI in cattle, sperm and maternal innate immunity have different effects when sperm hyperactivation occurs in the uterus and oviduct. Hyperactivated sperm regulated partially via TLR2 activation in the uterus, effectively penetrates the endometrial mucus, enters the uterine glands and enhances the triggering of the pro-inflammatory response. From the maternal immunity perspective, this is beneficial because it clears the uterine cavity from excess and dead sperm and prepares the endometrium for embryo implantation. However, from the sperm perspective, this is detrimental. These hyperactivated sperm in the uterus may miss the opportunity to enter and interact with the oviductal environment. On the other hand, the sperm that hyperactivates later in the oviduct, can detach from the oviductal epithelium, reach the COCs and penetrate the cumulus cell matrix, which is essential for successful fertilization. Altogether, hyperactivation in either the uterus or oviduct serves as a double-edged sword for sperm and maternal innate immunity in the context of fertility.

Sperm hyperactivation in the FRT contributes to fertility in different ways. Of note, the location and timing of hyperactivation determine how sperm influence fertility. However, this review has identified some gaps in the present knowledge on the role of sperm hyperactivation in the uterus and oviduct. It is unknown if any physiological stimuli in the FRT induce sperm to hyperactivate. It is also necessary to investigate the role of sperm hyperactivation in the uterus during natural mating. Therefore, there may be other ways that sperm hyperactivation affects bovine fertility that have not been discovered yet.
